# Risk Factors for Injury in CrossFit^®^—A Retrospective Analysis

**DOI:** 10.3390/ijerph20032211

**Published:** 2023-01-26

**Authors:** Sebastian Szajkowski, Michał Dwornik, Jarosław Pasek, Grzegorz Cieślar

**Affiliations:** 1 Faculty of Medical Sciences, Medical University of Mazovia in Warsaw, 8 Rydygiera St., 01-793 Warszawa, Poland; 2 Faculty of Health Sciences, Jan Długosz University in Częstochowa, 13/15Armii Krajowej St., 42-200 Częstochowa, Poland; 3 Department of Internal Medicine, Angiology and Physical Medicine, Faculty of Medical Sciences in Zabrze, Medical University of Silesia in Katowice, 15 Stefana Batorego St., 41-902 Bytom, Poland

**Keywords:** athletes, CrossFit, injures, sport activity, training process

## Abstract

CrossFit^®^ is a physical activity program and sport which is based on functional movements performed at high intensity and with high variability of exercises. It develops all motor skills. The study included 424 athletes (266 men and 158 women) from twelve centers in Poland, actively practicing CrossFit^®^ between 18 and 60 years of age. A questionnaire consisting of 25 questions was used, which was divided into four subsections concerning the characteristics of the sample, training routine, injuries, and information about environment. In total, 48% of respondents participating in the study suffered at least one injury during their entire training history. The injuries suffered most often involved shoulder joint and lumbar spine. Men were found to face a higher risk of injury than women, at 32.78% vs. 15.33% (*p* = 0.027). The shorter the training period, the smaller the number of injuries observed among the trainees. It was also noted that the shorter the training period, the lower the number of injuries that occurred (*p* = 0.041). An increase in the number of training sessions per week did not increase the incidence of injuries (*p* > 0.05). Performing isometric exercises during warm-up reduced the likelihood of injury during CrossFit^®^ training itself (*p* = 0.012). Training despite of concomitant acute pain had a significant adverse effect on the incidence of injuries (*p* = 0.002). The most common risk factors for injury in the CrossFit^®^ training process include, in particular: gender, training experience, and length of training sessions. Proper warm-up including isometric exercises and training conducted without accompanying pain symptoms reduces the risk of injury.

## 1. Introduction

CrossFit^®^ is a physical activity program and sport which is based on functional movements performed at high intensity and with high variability of exercises. It develops all motor skills such as: strength, power, speed, endurance, coordination, agility, and flexibility [1]. CrossFit^®^ workouts include elements of Olympic-style weightlifting, gymnastics, running, swimming, as well as functional and interval training [2]. A key feature of CrossFit^®^ exercises is scalability. Scalability refers not only to load progression, but also to modifications introduced to movements that require greater skill and flexibility. Many affiliated clubs promote another key feature of CrossFit^®^ that is purportedly the reason for CrossFit^®^’s effectiveness and popularity—the sense of community. Affiliated CrossFit^®^ members reported experiencing significantly more connectedness, friendship, and community affiliation compared to other sports. Research indicates that consistency contributes to adherence to exercise recommendations [3]. Classes are held in groups and are conducted by certified trainers. This type of training is attractive to those practising it and mobilizes them to exercise. There is also a competition factor that is helpful in achieving better results, but it can also be a factor that increases the risk of injury. The correctness of performing individual exercises is controlled by the trainer who, while conducting classes, adjusts the difficulty level of the exercises to the current abilities of the trainees. This way of conducting classes eliminates most irregularities in the technique of performing exercises and is the safest form of training [4].

CrossFit^®^ is a relatively young sport. The development of the training system has only been going on for 20 years. Since its introduction, studies on the exposure of training athletes to injuries have been published. The aim of some studies was to assess the risk of injury among trainees, due to the high intensity of exercise and high levels of metabolic stress and fatigue, which directly translate into poor exercise technique [5,6,7]. The results of epidemiological studies on CrossFit^®^ training do not indicate direct causes of injuries [8,9]. Many authors agree that a large number of repetitions, combined with high loads and high intensity, is the factor responsible for injuries most often affecting the shoulder area and lumbar spine [10]. A review of the literature in this field, however, indicates that the knowledge about preventing injuries in CrossFit^®^ is still insufficient [11]. Therefore, it is important to identify the most important factors that increase the risk of injury. Therefore, accurate data related to the frequency of injuries and the body regions they affect, in connection with many variable factors around training, are needed. Finding such compounds will be useful in designing the safest and least traumatic training sessions. The difficulty here is the specificity of CrossFit^®^ training, resulting from the combination of various, often opposing motor skills [7].

Aim of the study: The aim of the study was to evaluate the potential risk factors for injury that are present when practising CrossFit^®^ and examine the number and location of injuries.

## 2. Material and Methods

### 2.1. Participants

Twelve CrossFit^®^ affiliated clubs in Poland agreed to participate in the research. A total of 500 athletes training the sport were asked to participate in the research. Overall, 424 athletes (266 men and 158 women) completed the survey, achieving the completion rate of 84.8%. The inclusion criteria of the sample were: active contact as indicated by ticking the box pertaining to CrossFit^®^, Inc. (Washington, DC, USA), age in the range of 18–60 years, and had been practising CrossFit^®^ for a minimum of six months. The exclusion criteria were as follows: co-existence of chronic diseases, and refusal to participate in the study. After agreeing to participate in the research, participants signed a written informed consent form. All procedures were carried out in accordance with the principles of ethics standards of the Helsinki Declaration (1964) and were approved by the University’s Institutional Review Board (MUM/2022/11).

### 2.2. The Questionnaire

The survey was developed and used to collect data. The questionnaire contained 25 questions and was divided into the following information parts: (I) sample characteristics, (II) training routine, (III) injuries, (IV) additional information about environment. Some of the questions in the questionnaire were adapted from previous studies [6,12,13]. All questions contained in the questionnaire were assessed in terms of their content (adequacy and relevance of the questions asked) by a doctor, a physiotherapist and a certified CrossFit^®^ trainer. The purpose of the above assessment was to obtain answers indicating correctly validated content (content validity). The questionnaire contained instructions on how to correctly complete the individual sections. Respondents could request for help if they did not understand any of the questions. The questionnaire has been checked before submission for its correctness. The number of answers provided and the number of refusals to answer were totaled. The obtained data was entered into a spreadsheet. The severity of pain during training was measured using the Visual Analogue Scale (VAS). To define an injury, its definition was used in accordance with Mehrab M. et al. [9] as “any damage during sustained training that prevented the participant from training, working, or competing in any way and for any period of time”. Respondents had the opportunity to report all types of injuries they suffered during CrossFit training, taking into account the part of the body such injuries affected and their number.

### 2.3. Injuries Incidence

The injury incidence has been determined. It refers to the number of new cases of injury in the sample, noted during the CrossFit^®^ training. The measure of incidence is a proportion, which is expressed in percentages.

### 2.4. Incidence Rates

The incidence rate is the number of new cases of injury per population in a given period. It is expressed as the number of injuries per 1000 h of participation in sports. The equation of Chambers RB was used to calculate the incidence rate [14]. Incidence rates were calculated based on 52 weeks of training.

$$\mathrm{incidence}\;\mathrm{rate}=\frac{\left(\mathrm{No}.\;\mathrm{of}\;\mathrm{sports}\;\mathrm{injuries}/\mathrm{year}\right)\;\times \;1000}{\left(\mathrm{No}.\;\mathrm{of}\;\mathrm{participants}\right)\;\times \;\left(\mathrm{hours}\;\mathrm{of}\;\mathrm{participation}\;\mathrm{in}\;\mathrm{sports}\;/\mathrm{week}\right)\;\times \;\left(\mathrm{weeks}\;\mathrm{of}\;\mathrm{year}\right)}$$

### 2.5. Statistical Analysis

Statistical analysis was performed with the use of the Statistica 13 package (Statsoft, Kraków, Poland). The Shapiro–Wilk test was used to test the normality of data. Non-normal distributions of data were noted. The descriptive statistics is presented as median and inter-quartile range (IQR; Q1–Q3). The Mann–Whitney U test was used for quantitative variables to compare two unmatched groups of non-parametric data. The chi-squared test was used to test the unadjusted association of categorical variables. Logistic regression was used to evaluate adjusted associations. For the covariates that were included in the logistic regression model, odds ratios (OR) and 95% confidence intervals (CI) were estimated. Injury incidence is presented as proportions (%) based on the total number of surveys completed. The level of statistical significance was set at *p* < 0.05.

## 3. Results

### 3.1. Participants

Questionnaires obtained from 424 athletes were analyzed. The demographic profile of the study participants, divided into groups of injured (n = 204) and not injured (n = 220) participants, is presented in Table 1. The median age was 34 years, and the BMI was 25.1 (kg/m^2^). The median and inter-quartile range of the length of training experience in the Injured group was 3 (2–4) years, in the Not Injured group 2.5 (1–4) years. The difference observed was statistically significant (*p* = 0.002). The median and inter-quartile range of pain severity during exercise, measured with the use of VAS scale, amounted to 4 (3–5) points in the Injured group and 3 (1–4) points in the Not Injured group. The results differed between the groups at a statistically significant level (*p* = 0.000001). Differences in other parameters, i.e., the duration of the training session and the number of training sessions per week, were not statistically significant (*p* > 0.05).

### 3.2. Incidence of Injuries

Of the 424 participants in the study, 204 people (48.11%) reported some kind of injury which occurred while practising CrossFit^®^ over the entire training period, which amounted to an average of 3.05 ± 1.84 years. Table 2 shows the relationship between the frequency of injury and the variables characterizing the group of respondents. The chi-squared test shows a relationship between the occurrence of an injury and the following: gender, training experience, the history of practising sports before starting CrossFit^®^ training, performing isometric warm-up exercises, and training despite the presence of acute pain resulting from irritation of overloaded anatomical structures. Of all the men in the study, the majority (32.78%) suffered an injury during training. On the other hand, a minority (15.33%) of all surveyed women indicated an injury sustained while practising CrossFit^®^. Gender turned out to have a significant impact on the incidence of injury (*p* = 0.027). The shorter the training period, the smaller the number of injuries observed among the trainees. The largest number (16.08%) of people who did not report an injury had been training for 0.5–1.5 years, whereas the largest number of people (11.58%) who suffered an injury had been training for 5–10 years. The differences observed in the incidence or absence of injuries in relation to the length of the training period were statistically significant (*p* = 0.041). Differences in the incidence of injuries depending on the duration of the training session were close to the level of significance (*p* = 0.056). There was no significant effect of the number of training sessions per week on the incidence of injuries (*p* = 0.160). The vast majority of respondents (83.01%) had practised sports before starting CrossFit^®^ training (*p* = 0.048). Among these people, the percentage distribution of those who were injured and not injured while practising CrossFit^®^ was almost identical and amounted to (41.75%) vs. (41.27%), respectively. More people who were inactive before starting CrossFit training (16.98%) were not injured during practising it (10.61%) vs. (6.37%). A minority of the surveyed CrossFit^®^ trainees (37.5%) declared performing isometric exercises during the warm-up. In that group, more people (22.41%) did not have an injury than the portion for whom an injury caused a break in training (15.09%). The opposite distribution of results was observed among the majority of people (62.5%) who did not perform isometric exercises during the warm-up. In that case, more people reported being injured (33.02%) than those who did not (29.48%). Isometric exercises additionally performed during the warm-up had a significant impact on the frequency of injuries (*p* = 0.012). The majority (22.17%) of those who exercised despite experiencing acute pain other than Delayed Onset Muscle Soreness (DOMS) (38.44%) sustained an injury during CrossFit^®^ training. Conversely, fewer respondents among non-training people declared an injury when experiencing acute pain resulting from irritating diseased tissues, (25.94%) vs. (35.16%), respectively. The fact of training despite the coexisting acute pain had a significant impact on the frequency of injuries (*p* = 0.002). There was no relationship between the incidence of injuries and other variables presented in Table 2 (*p* > 0.05).

Longer history of experience with CrossFit^®^ training increased the odds of being injured (OR = 1.139; *p* = 0.046). Stopping training while experiencing acute pain other than DOMS reduced the risk of injury by a factor of 0.5 (OR = 0.54; *p* = 0.004). Performing isometric exercises during warm-up reduced the likelihood of injury (OR = 0.563; *p* = 0.008) during CrossFit^®^ training. Males had increased odds of being injured (OR = 0.515; *p* = 0.011). Other analyzed variables presented in Table 3 did not have a statistically significant effect (*p* > 0.05) on the risk of injury that may occur while practising CrossFit^®^.

In total, 115 out of the 204 people surveyed reported that during their entire training experience (average 3.3 ± 1.81 years) they suffered injuries that affected more than one part of the body. The total number of injuries in the analyzed research material was 392 (1.92 injuries/person on average). Of the 81 respondents who reported an injury in the last 12 months, 36 people reported more than one injury. In total, 117 injuries were recorded over the last year (average: 1.44 injuries/person). Table 4 presents the frequency of all injuries and the incidence rate of respective injured body part (per 1000 athlete training hours). As noted during the year of observation, injuries most often concerned the shoulder joint (24/117) and lumbar spine (23/117). The hip joint was indicated by the respondents as the least likely place of injury (2/117). The injury rate ranged from 0.06 for the hip to 0.73 for the shoulder per 1000 h of training. In total, 15 (7.35%) out of the 204 people injured during CrossFit^®^ training were treated surgically. In 8 cases, the surgery was related to the knee joint, in 3 cases to lumbar spine discopathy, in 3 cases to inguinal hernia, and in 1 case to the reconstruction of the Achilles tendon. The average duration of a break in training due to injury was 2.59 ± 4.13 months.

## 4. Discussion

In recent years, CrossFit^®^ has become a very popular form of training. Many people compete in various sport competitions [15]. Mehrab et al. [9] noted that this is a young sport and recreation sport discipline, and therefore the knowledge about the risk factors for injuries while practising it is not well-established. Many studies also indicate that the injury rate in CrossFit^®^ is in the range of: 1.9–3.1/1000 h of training and does not differ from other popular sports, such as Olympic weightlifting: 2.4–3.3/1000 h, rugby: 3–4.2/1000 h, football: 4.22/1000 h, or gymnastics: 3.1/1000 h [11,16,17,18], and is lower than in bodybuilding: 0.2–1/1000 h [19].

In our study, 48% of the respondents suffered at least one injury during their entire training experience period (min. 0.5–max. 10 years). The results of other studies indicate that this is relatively not high, because every person practising sports regularly is at risk of suffering some kind of injury. There are many factors that increase the risk of injury: older age, repetitive injuries, or training routine. One can only minimize the negative impact of modifiable factors related to the training methodology. Thus, the total injury rate of 48% is not excessive and results from the short training experience of the respondents, 3 years on average, and the methodology of conducting training in an organized manner. The specificity of CrossFit^®^ training consist in the implementation of a training unit assigned to a given day—WOD (Workout Of the Day) under the strict supervision of a trainer [20,21].

In Claudino’s et al. meta-analysis, including 204 publications, CrossFit^®^ injury rates range from 19% to 74% [22]. The percentage of injuries in our study (48%) is therefore in the middle of that range, as in the largest study based on meta-analysis of 280 publications (average: 35.3%, range from 12.8% to 73.5%) [16].

Our study confirmed that body parts which are the most susceptible to injury that can occur while practising CrossFit^®^ include: the shoulder joint and lumbar spine. The largest study we found based on the material and data of 449 people training CrossFit^®^ was conducted by Mehrab et al. [9]. In their study, the overall trauma rate was 56.1% vs. 48% in our study. The percentage distribution of the number of injuries among specific parts of the body compared to our study was as follows: shoulder joint: 28.7% vs. 20.51%, lumbar spine: 15.8% vs. 19.65%, knee joint: 8.3% vs. 12.82%. Similar results were also obtained by other researchers [6,7,10,23,24,25,26]. The lowest rate of injuries in our study involved the hip joint: 1.7% vs. 3%. This is also confirmed by the research of Rodriguez et al. and Cheng et al. [16,27]. Therefore, analyzing our own research and that of other researchers, there is no doubt concerning the structures of the musculoskeletal system which are most exposed to injuries. Such knowledge allows one to introduce preventive measures during warm-up, training itself, and post-training activities. The above-mentioned shoulder joint and lumbar spine are most often injured during CrossFit^®^ weightlifting. Research confirms the highest level of trauma affecting those body parts in Olympic weightlifting, which are very often used in exercises performed in the same form during CrossFit^®^ training [8,28,29].

Our research also shows that to reduce injury rates in the CrossFit^®^ training process, several factors must be considered in parallel. These include in particular: gender, training experience, previous sports activity, proper warm-up including isometric exercises, and training without pain symptoms. In our study, men who usually trained more intensely and under bigger loads also suffered injuries more often. This is also confirmed by observations by Sugimoto D. et al. [30].

Particular attention should be paid to training experience. It turns out that statistically significantly more injuries occur in the advanced period of CrossFit^®^ training, which entails over 5 years of training experience. This may be due to the fact that in the initial period all trainings take place in groups under the supervision of a trainer. In the later years of training, athletes try much more technically difficult exercises combined with greater intensity, often without the constant supervision of a trainer. Many people with longer training experience also participate in competitions. Similar observations can be found in the studies by Feito et al. and Alekseyev et al., which showed that the cause of injury is not only related to the increasing training experience [7,15]. The frequency of injuries is also correlated with incorrect increase in load and training progression, according to the theory proposed by Gabbett—“Training-Injury Prevention Paradox” [31].

Another important factor to consider when planning one’s CrossFit^®^ workouts is not having practised any sport before. On the one hand, practising sports earlier may result in experience in injury prevention, on the other hand, it is an additional burden, with the heritage of injuries already sustained. The results of our study indicate a lower risk of injury when practising CrossFit^®^ for people who have not trained before. This is explained by the fact that they are less likely to have suffered an injury in the past. Each trauma increases the risk of another one, due to pathological structural changes in damaged tissues [20].

The risk factors for injury presented above: training experience and sports activity before practising CrossFit^®^ can be effectively influenced through education and proper interpretation of pain symptoms in trainees. The factors that play an important role in the prevention of injuries excluding activity in sports for a long period of time include: proper warm-up and the absence of pain symptoms during training [20,32].

According to our research, a proper and effective warm-up should include isometric exercises. This warm-up component statistically significantly reduces the likelihood of injury. Isometric exercises lead to temporary reduced blood flow, as a result of the constant pressure of muscle tissue on blood vessels. After the cessation of isometric tensions, blood vessels dilate, increasing the blood supply and flexibility of muscles [33]. Similarly, constant long-term pressure of connective tissue—tendons—during isometric contraction can improve their biomechanical properties in the mechanism of improving blood supply and, consequently, increasing hydration. This contributes to the prevention of injuries [34]. The mechanism of improving blood circulation based on the stimulation of nitric oxide (NO) production exerted by physical exercise is described by Green and Smith [35]. The connective tissue of the musculoskeletal system has a much greater hardness than muscle tissue and is less vascularized than the latter. This predisposes to injuries and may cause poorer healing of the resulting damage. Increased blood circulation within these structures during the warm-up effectively reduces the risk of injury, and prevents the accumulation of changes due to overload and micro-injuries—DOMS and Delayed-Onset Soft-tissue Stiffness (DOSS). It also improves deformation properties of connective tissue, thus increasing its strength [36,37,38]. The legitimacy of performing isometric exercises during warm-up as a means of preventing injuries is also confirmed by the studies of Skurvydas et al. and Kubo et al. [39,40].

Another statistically significant factor in preventing injuries that was confirmed in our study is the performance of exercises without accompanying pain symptoms. Pain is always a sign of tissue damage, thus conducting training with co-occurring/coexisting pain symptoms always aggravates the original injury [36,37]. In the training process, pain may and should occur, for example, DOMS and DOSS. In such cases, training should be discontinued until the pain subsides. Depending on the degree of training, the symptoms of DOMS and DOSS should disappear after 36–72 h. Trainers should be educated in distinguishing between different types of pain. Athletes should be able to differentiate “soreness” from acute pain, which is the basis for stopping training, and often also for the implementation of treatment [36,37,41]. The results of our study prove a statistically significant, preventive effect of training without concomitant acute pain symptoms on the occurrence of injuries. This is also confirmed by the research conducted by Johnston et al. [42].

Among the results of our research, there are two risk factors for injury, the analysis of which did not reach the level of statistical significance, but which in our opinion require comments and discussion. The first one is the length of training sessions. The duration of a training unit may cause excessive fatigue and force errors during exercise, it can also increase the overall overload of musculoskeletal tissues, which in turn may lead to injury. Thus, the longer the training session, the greater the susceptibility to injury, which is confirmed by the results of our research. Another risk factor for injury, without confirmed statistical significance in this study, is the number of training sessions per week. According to our analyses, more training sessions per week do not necessarily increase the incidence of injuries, as in Alekseyev’s study. The explanation of this phenomenon can be the experience of trainees [15].

It should be emphasized that adhering to the principle of stopping training when experiencing acute pain that may be a symptom of structural tissue damage can significantly reduce the rate of serious injuries associated with practising CrossFit^®^. Isometric exercises performed in addition to warm-up can further reduce the risk of injury, thus ranking CrossFit^®^ below the average for sport disciplines.

## 5. Limitations of the Study

A limitation of our study was the mere use of a questionnaire to retrospectively assess injury risk factors among amateur CrossFit^®^ athletes. Following the implementation of strategies to reduce the rate of injury clearly evident from our work, further prospective studies are necessary to achieve the goal indicated.

## 6. Conclusions

This study provides a valuable contribution to a better understanding of the causes of CrossFit^®^ injuries. The results of the conducted research allow us to draw conclusions and formulate practical guidelines on the basis of which, if followed during CrossFit^®^ training, the percentage of injuries can be significantly reduced. Isometric exercises should be an important part of trainees’ warm-up. It is advised to stop exercising if one experiences acute pain. Caution should be exercised especially by men with long training experience. Particular attention should be paid to exercises that strongly involve shoulder joints and lumbar spine. More research is required to improve our understanding of how to prevent injuries when practising CrossFit^®^.

The authors encourage the entire sports community: certified trainers as well as people training CrossFit^®^ both recreationally and professionally to take into account the above conclusions and follow the guidelines both in training process and during CrossFit^®^ competitions.

## Figures and Tables

**Table 1:** Demographic profile per injury.

	Total (n = 424)	Injured (n = 204)	Not Injured (n = 220)	* *p*-Value
	Median (IQR)	Median (IQR)	Median (IQR)	
Age (years)	34 (29–40)	34 (29–39)	34 (29–40)	0.774
Height (cm)	176 (170–182)	176 (170–182)	175 (168–182)	0.497
Weight (kg)	80 (67–88.9)	80 (68–88.5)	79.5 (65–88.9)	0.482
BMI (kg/m^2^)	25.1 (22.98–27.13)	25.37 (23.14–27.04)	24.87 (22.63–27.16)	0.513
Experience in CrossFit training (years)	3 (1.5–4)	3 (2–4)	2.5 (1–4)	**0.002**
Weekly participation in CrossFit training (days)	4 (3–5)	4 (3–5)	4 (3–5)	0.874
Length of training sessions (hours)	1 (1–1.5)	1 (1–1.5)	1 (1–1.5)	0.112
Training despite of pain—other than (DOMS) (VAS-scale)	3 (2–5)	4 (3–5)	3 (1–4)	**<0.001**

* Mann–Whitney U-test. *p*-value in bold mean statistical significance.

**Table 2:** Relationship between injuries incidence and respondents’ characteristics.

	Total = 424 (100)	Injured = 204 (48.11)	Not Injured = 220 (51.89)	* *p*-Value
	n (%)	n (%)	n (%)	
Gender				
Male	266 (62.73)	139 (32.78)	127 (29.95)	**0.02** **7**
Female	158 (37.26)	65 (15.33)	93 (21.93)	
Age (years)				
18–29	117 (27.59)	52 (12.26)	65 (15.33)	0.501
30–39	200 (47.16)	102 (24.06)	98 (23.11)	
40–56	107 (25.23)	50 (11.79)	57 (13.44)	
BMI (kg/m^2^)				
Normal weight (18.5–24.99)	205 (48.34)	94 (22.17)	111 (26.18)	0.368
Overweight (25–29.99)	219 (51.65)	110 (25.94)	109 (25.71)	
Experience in CrossFit^®^ training (in years)				
0.5–1.5	108 (25.47)	39 (9.22)	68 (16.08)	**0.041**
2–2.5	87 (20.51)	43 (10.17)	44 (10.4)	
3–3.5	76 (17.92)	36 (8.51)	40 (9.46)	
4–4.5	65 (15.33)	37 (8.75)	28 (6.62)	
5–10	88 (20.75)	49 (11.58)	39 (9.22)	
Length of training sessions (hours)				
0.5–1	263 (62.02)	117 (27.59)	146 (34.43)	0.056
1.5–3	161 (37.97)	87 (20.52)	74 (17.45)	
Weekly participation in CrossFit^®^ training (days)				
1–3	159 (37.5)	73 (17.22)	86 (20.28)	0.160
4	131 (30.89)	72 (16.98)	59 (13.92)	
5–10	134 (31.6)	59 (13.92)	75 (17.69)	
Participation in sports before taking to CrossFit^®^				
Yes	352 (83.01)	177 (41.75)	175 (41.27)	**0.04** **8**
No	72 (16.98)	27 (6.37)	45 (10.61)	
Sport activity outside CrossFit^®^				
Yes	273 (64.38)	133 (31.37)	140 (33.02)	0.738
No	151 (35.61)	71 (16.75)	80 (18.87)	
Warm-up included in workouts				
Yes	408 (96.22)	195 (45.99)	213 (50.24)	0.507
No	16 (3.77)	9 (2.12)	7 (1.65)	
Cool-down included in workouts				
Yes	238 (56.13)	117 (27.59)	121 (28.54)	0.626
No	186 (43.86)	87 (20.52)	99 (23.35)	
Stretching included in warm-up				
Yes	342 (80.66)	162 (38.21)	180 (42.45)	0.531
No	82 (19.33)	42 (9.91)	40 (9.43)	
Stretching included in cool-down				
Yes	253 (59.66)	117 (27.59)	136 (32.08)	0.349
No	171 (40.33)	87 (20.52)	84 (19.81)	
Isometric exercises included in warm-up				
Yes	159 (37.5)	64 (15.09)	95 (22.41)	**0.012**
No	265 (62.5)	140 (33.02)	125 (29.48)	
Training despite of pain—other than (DOMS)				
Yes	163 (38.44)	94 (22.17)	69 (16.27)	**0.00** **2**
No	261 (61.55)	110 (25.94)	151 (35.16)	
Routine physiotherapy exposure				
Yes	161 (37.97)	84 (19.81)	77 (18.16)	0.190
No	263 (62.02)	120 (28.3)	143 (33.73)	
Professional work				
White collars	337 (79.48)	157 (37.03)	180 (42.45)	0.216
Blue collars	87 (20.51)	47 (11.08)	40 (9.43)	
Participation in CrossFit^®^ competitions				
Competitor	58 (13.67)	30 (7.08)	28 (6.6)	0.554
Non-Competitor	366 (86.32)	174 (41.04)	192 (45.28)	

* Chi-squared test. *p*-value in bold mean statistical significance.

**Table 3:** Logistic regression analysis of injury risk factors.

Variable	Odds Ratio	(95% CI)	*p*-Value
Gender	0.515	0.308–0.861	**0.011**
Age	1.006	0.976–1.037	0.662
BMI	0.918	0.831- 1.014	0.094
Experience in CrossFit^®^ training	1.139	1.001–1.295	**0.046**
Participation in sport before starting with CrossFit^®^	1.615	0.895–2.913	0.110
Sport activity outside CrossFit^®^	0.904	0.580–1.411	0.659
Weekly participation in CrossFit^®^ training	0.946	0.801–1.117	0.516
Length of training sessions	1.213	0.726–2.027	0.459
Participation in CrossFit^®^ competitions	0.679	0.315–1.460	0.321
Professional work	1.157	0.692–1.933	0.576
Training despite of pain—other than (DOMS)	0.54	0.354–0.823	**0.004**
Routine physiotherapy exposure	1.194	0.774–1.842	0.420
Warm-up included in workouts	0.999	0.963–1.037	0.988
Cool-down included in workouts	1.621	0.907–2.896	0.102
Stretching included in warm-up	0.841	0.489–1.445	0.530
Stretching included in cool-down	0.621	0.350–1.099	0.101
Isometric exercises included in warm-up	0.563	0.367–0.863	**0.008**

*p*-value in bold mean statistical significance.

**Table 4:** Frequency, percentage, and incidence rate of injured body part.

Body Part	Frequency	Percent	Incidence/1000 Athlete Training Hours
shoulder	24	20.51	0.73
lower back/lumbar spine	23	19.65	0.7
knee	15	12.82	0.46
arm muscles	12	10.25	0.36
wrist/hand	12	10.25	0.36
ankle/foot	6	5.12	0.18
elbow	6	5.12	0.18
forearm muscles	4	3.41	0.12
thigh muscles	4	3.41	0.12
neck/cervical spine	3	2.56	0.09
upper back/thoracic spine	3	2.56	0.09
shank muscles	3	2.56	0.09
hip	2	1.7	0.06

## Data Availability

The datasets used and/or analyzed during the current study are available from the corresponding author on reasonable request.

## Abbreviations

BMI	Body Mass Index
DOMS	Delayed Onset Muscle Soreness
DOSS	Delayed Onset Softissue Stiffness
VAS	Visual Analogues Scale
